# *Angelica dahurica* Extracts Improve Glucose Tolerance through the Activation of GPR119

**DOI:** 10.1371/journal.pone.0158796

**Published:** 2016-07-08

**Authors:** Eun-Young Park, Eung-Hwi Kim, Chul-Young Kim, Mi-Hwi Kim, Jin-Seung Choung, Yoon-Sin Oh, Hong-Sub Moon, Hee-Sook Jun

**Affiliations:** 1 College of Pharmacy, Mokpo National University, Muan-gun, Jeonnam, 534-729, Republic of Korea; 2 Lee Gil Ya Cancer and Diabetes Institute, Gachon University, 7-45 Songdo-dong, Yeonsu-ku, Incheon, 406-840, South Korea; 3 College of Pharmacy, Hanyang University, Ansan, Kyeonggi, 426-791, Republic of Korea; 4 Gachon Medical Research Institute, Gil Hospital, Incheon, South Korea; 5 College of Pharmacy and Gachon Institute of Pharmaceutical Science, Gachon University, 7-45 Songdo-dong, Yeonsu-ku, Incheon, 406-840, South Korea; University of Bremen, GERMANY

## Abstract

G protein-coupled receptor (GPR) 119 is expressed in pancreatic β-cells and intestinal L cells, and is involved in glucose-stimulated insulin secretion and glucagon-like peptide-1 (GLP-1) release, respectively. Therefore, the development of GPR119 agonists is a potential treatment for type 2 diabetes. We screened 1500 natural plant extracts for GPR119 agonistic actions and investigated the most promising extract, that from *Angelica dahurica* (AD), for hypoglycemic actions *in vitro* and *in vivo*. Human GPR119 activation was measured in GeneBLAzer T-Rex GPR119-CRE-bla CHO-K1 cells; intracellular cAMP levels and insulin secretion were measured in INS-1 cells; and GLP-1 release was measured in GLUTag cells. Glucose tolerance tests and serum plasma insulin levels were measured in normal C57BL6 mice and diabetic db/db mice. AD extract-treated cells showed significant increases in GPR119 activation, intracellular cAMP levels, GLP-1 levels and glucose-stimulated insulin secretion as compared with controls. In normal mice, a single treatment with AD extract improved glucose tolerance and increased insulin secretion. Treatment with multiple doses of AD extract or n-hexane fraction improved glucose tolerance in diabetic db/db mice. Imperatorin, phellopterin and isoimperatorin were identified in the active fraction of AD extract. Among these, phellopterin activated GPR119 and increased active GLP-1 and insulin secretion *in vitro* and enhanced glucose tolerance in normal and db/db mice. We suggest that phellopterin might have a therapeutic potential for the treatment of type 2 diabetes.

## Introduction

Type 2 diabetes mellitus is a common metabolic disorder which is increasingly prevalent throughout the world [1]. Type 2 diabetes is characterized by insulin resistance and hyperglycemia [2–5]. Various pathologies, including defective insulin secretion from pancreatic β-cells, inadequate hepatic glucose production and peripheral insulin resistance, are involved in disease development, and many therapeutic approaches have been attempted to treat this metabolic syndrome [1, 6].

Recently, incretin-based therapies such as glucagon-like peptide-1 (GLP-1) analogues and dipeptidyl peptidase-4 inhibitors have been used for treating diabetic patients [7], but these drugs have shown clinically limited effects. For instance, GLP-1 analogs require intraperitoneal injections, and the efficacy of dipeptidyl peptidase-4 inhibitors is modest as their action is dependent on endogenous GLP-1 [8].

G-protein-coupled receptor 119 (GPR119) is a member of the class A, G protein-coupled receptor family. GPR119 is highly expressed in pancreatic β-cells and intestinal endocrine L-cells [9–11]. The activation of GPR119 promotes glucose-stimulated insulin secretion and GLP-1 release, thus GPR119 is considered to be a potential therapeutic target for the treatment of type 2 diabetes [2, 11, 12].

Herbal extracts have received increasing attention as complementary and alternative medicines for the treatment of diabetes. *Angelica dahurica Bentham et Hooker f*. (Umbelliferae, AD) is a herbal medicine which has been widely used as traditional medicine in Korea, China, and Japan to treat colds, headaches, and toothaches. It was reported that active compounds derived from AD, including coumarin (PubChem CID:323), phellopterin (PubChem CID:98608), isoimperatorin (PubChem CID:68081), imperatorin (PubChem CID:10212), oxypeucedanin (PubChem CID:33306), byakangelicin (PubChem CID:10211), and pimpinellin (PubChem CID:4825), have anti-inflammatory, anti-mutagenic, anti-microbial, anti-tumor, and anti-oxidant activities [13–16]. It has been proposed that furocoumarin (PubChem CID:6199) derivatives from AD roots have anti-diabetic effects through retinoic aicd receptor-α activation [17]. In this study, we investigated the effects of AD extracts on GLP-1 and insulin secretion by glucose challenge and their GPR119 agonist effects *in vitro* and *in vivo*.

## Materials and Methods

### Preparation of crude extract

AD roots were purchased from a Kyungdong oriental herbal market (Seoul, Korea). A voucher specimen was deposited in the Herbarium of the College of Pharmacy, Hanyang University (HYU-AD-001). The dried roots of AD (200 g) were extracted at room temperature with methanol (500 mL × 3) using an ultrasonic apparatus, then concentrated *in vacuo* to yield 27.5 g of crude extract. The concentrated residue was resuspended in 1,000 mL water and was successively partitioned three times with 1,000 mL of *n*-hexane, ethyl acetate and *n*-butanol; 4.7 g of *n*-hexane extract, 1.4 g of ethyl acetate extract, 4.8 g of *n*-butanol extract and 16 g of water extract were obtained.

### HPLC analysis of crude extract

The crude AD extracts were analyzed by HPLC using an Agilent 1200 HPLC system with an INNO C18 column (250 mm × 4.6 mm, 5 μm, Youngjin Biochrom, Korea) at a flow-rate of 1 mL/min of mobile phase controlled by binary pumps at 40°C. The mobile phase was a linear gradient of acetonitrile with 0.1% formic acid (A) and water with 0.1% formic acid (B): 0–20 min, 47–50% A; 20–35 min, 50–70% A; 35–40 min, 75–100% A. The injection volume of the crude total extract and *n*-hexane fraction was 10 μL. The effluent was monitored at 254 nm. The respective retention times of the three major peaks were 19.1, 22.3 and 26.2 min.

### Cell culture

GeneBLAzer^®^ T-REx^™^ GPR119-CRE-*bla* CHO-K1 cells (Invitrogen, Carlsbad, CA, USA) were cultured in DMEM high glucose with 10% fetal bovine serum (Gibco BRL, NY, USA), 100 U/mL penicillin, 100 μg/mL streptomycin, non-essential amino acids 0.1 mM, HEPES (pH 7.3) 25 mM, Zeocin^™^ 100 μg/mL, and hygromycin 600 μg/mL. INS-1 rat insulinoma cells were maintained in RPMI-1640 (Gibco BRL, NY, USA) supplemented with 11 mM glucose, 10% fetal bovine serum, 100 U/mL penicillin, 100 μg/mL streptomycin, 50 μM beta-mercaptoethanol and 1 mM sodium pyruvate. GLUTag mouse enteroendocrine L cells were cultured in DMEM (Gibco BRL, NY, USA) supplemented with 5.5 mM glucose, 10% fetal bovine serum, 100 U/mL penicillin and 100 μg/mL streptomycin.

### Animals

Eight-week-old male C57BL6 wild-type and db/db mice (C57BL/KsJ background) were obtained from Orient Bio Inc. (Gyeonggi-do, Korea) and the Korea Research Institute of Bioscience and Biotechnology (Daejeon, Korea) respectively. Mice were maintained under specific pathogen-free conditions in a temperature-controlled room (23 ± 1°C) with a 12-h light/dark cycle and *ad libitum* access to food and water at the Animal Care Center, Lee Gil Ya Cancer and Diabetes Institute, Gachon University. This study was carried out in strict accordance with the recommendations in the Guide for the AAALAC. All animal experiments were approved by the Institutional Animal Care and Use Committee of the Lee Gil Ya Cancer and Diabetes Institute, Gachon University.

### GPR119 reporter assay

GeneBLAzer^®^ T-REx^™^ GPR119-CRE-*bla* CHO-K1 cells contain the human G-protein receptor 119 (GPR119), which is stably integrated into the CRE-*bla* CHO-K1 cell line. CHO-K1 cells also contain a β-lactamase reporter gene under control of the cAMP response element. In brief, GeneBLAzer^®^ T-REx^™^ GPR119-CRE-*bla* CHO-K1 cells were seeded in 96-well culture plates at 2 × 10^4^ cells per 80 μL. The next day, cells were treated 20 μL of assay media (Cat No. 10569) containing various concentrations of AD extract or compounds. After 5 h of incubation, LiveBLAzer^™^-FRET B/G Substrate (Invitrogen, Carlsbad, CA, USA) was added, and plates were incubated in the dark at room temperature for 2 h. Fluorescence emission values were measured at 460 nm (blue) and 530 nm (green) using a VICTOR3 microplate reader (PerkinElmer Life Sciences, Waltham, MA, USA) and the blue/green emission ratio was calculated for each well.

### cAMP assay

GLUTag and INS-1 cells were seeded in 12-well plates at a density of 1 × 10^5^ cells/well and incubated for 24 h. The cells were washed with PBS and then incubated in Krebs-Ringer bicarbonate buffer (KRB) (116 mM NaCl, 1.8 mM CaCl_2_•2(H_2_O), 0.8 mM MgSO_4_•7(H_2_O), 5.4 mM KCl, 1 mM NaH_2_PO_4_•2(H_2_O), 26 mM NaCHO_3_, and 0.5% BSA, pH 7.4) containing AD extract (100 μg/mL) for 30 min. Cells were harvested, and the total lysate was extracted using 0.1N HCl. The concentration of cAMP was measured with a cAMP ELISA kit (Enzo Life Sciences, Farmingdale, NY, USA) according to the manufacturer’s protocol.

### Measurement of active GLP-1 secretion

GLUTag cells were seeded (1 × 10^5^ cells / well) into 24-well culture plates and incubated for 24 h. The cells were washed with twice with KRB buffer (0.114 M NaCl, 4.7 mM KCl, 1.2 mM KH_2_PO_4_, 1.16 mM MgSO_4_•7(H_2_O), 2.5 mM CaCl_2_, 25.5 mM NaHCO_3_, 20 mM HEPES, 0.2% BSA, pH = 7.2) and incubated in KRB buffer containing 10 mM glucose, 0.2 μM dipeptidyl peptidase-4 inhibitor and AD extracts for 30 min. The supernatants were collected and GLP-1 secretion was determined using the GLP-1 (Active 7–36) ELISA kit (Alpco Diagnostics, NH, USA).

### Glucose-stimulated insulin secretion

INS-1 cells were washed and incubated in KRB buffer. Cells were pre-incubated in KRB buffer (0.1 mM glucose) with AD extracts for 2 h and then incubated in KRB buffer supplemented with 3 or 17.5 mM glucose for an additional 90 min at 37°C, 5% CO_2_. The insulin levels were measured using a rat ultrasensitive ELISA kit (Alpco Diagnostics, NH, USA), according to manufacturer’s instruction. The insulin content was normalized to the protein content of the corresponding cell lysates.

### Oral glucose tolerance tests and measurement of serum insulin levels

Mice were fasted for 14 h and then AD extracts or single compounds were given by oral intubation. After 30 min, a glucose solution (2 g/kg body weight in PBS) was administered orally and blood samples were collected from the tail vein at 0, 5, 10, 15, 30 60, 90 and 120 min. Blood glucose levels were measured using a glucometer (LifeScan, Inc. One Touch Ultra). Serum insulin levels were determined at 0, 5, 10 and 15 min using a mouse insulin ultrasensitive ELSIA kit (Alpco Diagnostics, NH, USA).

### Treatment of db/db mice with AD extracts or n-hexane fractions

At 9–10 weeks of age, db/db mice were randomly divided into three treatment groups (n = 10–11 in each group): phosphate buffered saline (PBS), methanol extract of AD (Total), and n-hexane fraction of AD (n-hexane). Total extract or n-hexane fraction (300 mg/kg in PBS) was administered daily by oral intubation for 3 weeks. Blood glucose levels, body weight, and food consumption were measured weekly. We daily monitored for signs of disease and physical and behavioral abnormalities. All mice remained alive until experimental endpoint and were sacrificed by CO2 inhalation.

### Purification of compounds from the n-hexane fraction of AD

The n-hexane fraction of AD extracts was further purified by preparative HPLC. Preparative HPLC separation was carried out by injection 500 μL of *n*-hexane extract onto an Inno C18 column (20 x 250 mm, 5 μm, YoungJin Biochrom, Korea). The mobile phase consisted of methanol containing 0.1% formic acid and water containing 0.1% formic acid, which was programmed as follows: 0–40 min, 40–100% A; 40–50 min, 100% A. The flow rate was 10 mL/min and the chromatogram was monitored at 254 nm. Preparative HPLC led to purification of imperatorin (161.5 mg), phellopterin (60.5 mg) and isoimperatorin (157.3 mg). The structural identification of isolated compounds 1–3 was carried out by ESI-MS, ^1^H and ^13^C NMR. NMR spectra were obtained in CDCl_3_ using an AVANCE III 400 spectrophotometer (Bruker, Germany). ESI-MS data were measured on an Advion Expression CMS system (New York, USA). ESI-MS conditions were as follows: positive ion mode; mass range, m/z 100–1000; capillary temperature, 250°C; capillary voltage, 150 V; source voltage offset, 30; source voltage span, 10; source gas temperature, 150°C; ESI voltage, 3,500 V. The HPLC chromatograms and ^1^H NMR spectra of isolated compounds 1–3 were added in S1–S4 Figs.

### Statistical analyses

Data are presented as mean ± SE. The significance of differences was analyzed by one-way ANOVA with the post-hoc Duncan procedure using SPSS ver. 10.0 (SPSS Inc.) The value of statistical significance was set at p<0.05.

## Results

### Identification of AD extracts with GPR119 agonistic activity

**Fig 1:**
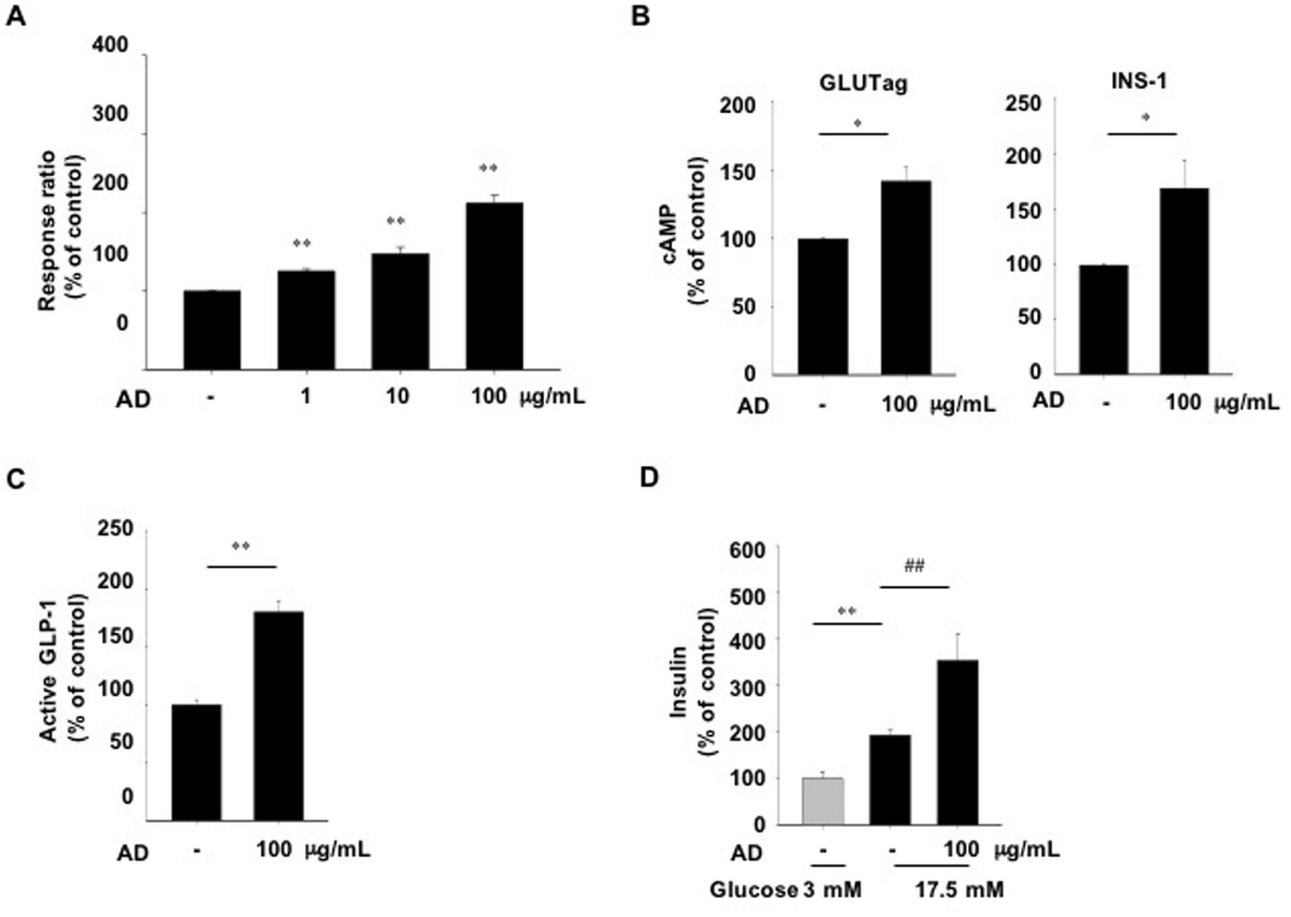
Agonistic and biological activities of AD extract *in vitro*. (A) β-lactamase activity as a reporter was measured in the GPR119-CRE-*bla* CHO-K1 cell line treated with the indicated concentrations of AD extract for 5 h. (B) Intracellular cAMP levels were measured in GLUTag and INS-1 cells treated with AD extract for 30 min. (C) Active GLP-1 secretion from GLUTag cells treated with AD extract for 30 min. (D) Glucose-stimulated insulin secretion in INS-1 cells treated with AD extract for 2 h in the presence of low (L, 3 mM) or high (H, 17.5 mM) glucose. AD extract dissolved in dimethylsulfoxide (DMSO). n = 3-4/group. Values are means ± SE. *p<0.05, **p<0.01 compared with vehicle treatment (DMSO), ##p<0.01 compared with vehicle treated cells in the presence of high glucose.

We screened a library of ~1,500 natural plant extracts for GPR119 agonistic activity using the GPR119-CRE-*bla* CHO-K1 reporter cell line. Several hits were identified for activation of the β-lactamase luciferase reporter expression in GPR119-CRE-*bla* CHO-K1 cells (data not shown). We chose AD methanol extracts as a potent candidate for the GPR119 agonist. To confirm that the AD extracts have GPR119 agonistic activity, we first measured the β-lactamase luciferase activity after treatment with different concentrations of AD extracts. The reporter activity was increased in a dose-dependent manner, suggesting that intracellular cAMP production was stimulated by AD extract treatment (Fig 1A). As GPR119 is coupled to the Gαs protein and activated to induce cAMP production, we directly measured cAMP accumulation in an enteroendocrine cell line (GLUTag cells) and a pancreatic β-cell line (INS-1 cells), which are known to express GPR119 [10]. We found that cAMP levels were significantly enhanced both in GLUTag and INS-1 cells after treatment with 100 μg/mL of AD extracts (Fig 1B), the dose which showed the highest activity in the reporter cell line. Consistent with these results, active GLP-1 levels were significantly increased in GLUTag cells by AD extract treatment (Fig 1C). Agonists of GPR119 stimulate insulin secretion from pancreatic β-cells [9]. To determine the direct effects of AD extracts on pancreatic β-cells, we measured glucose-stimulated insulin secretion in INS-1 cells. Treatment with AD extracts significantly increased insulin secretion in INS-1 cells exposed to 17.5 mM glucose. AD treatment slightly simulated the secretion of insulin at 3 mM glucose, but this was not statistically significant (data not shown). These data suggest that the AD extracts have an agonistic action for GPR119.

### Effect of AD extracts on glucose tolerance and glucose-stimulated insulin secretion after a single administration in C57BL6 and *db/db* mice

**Fig 2:**
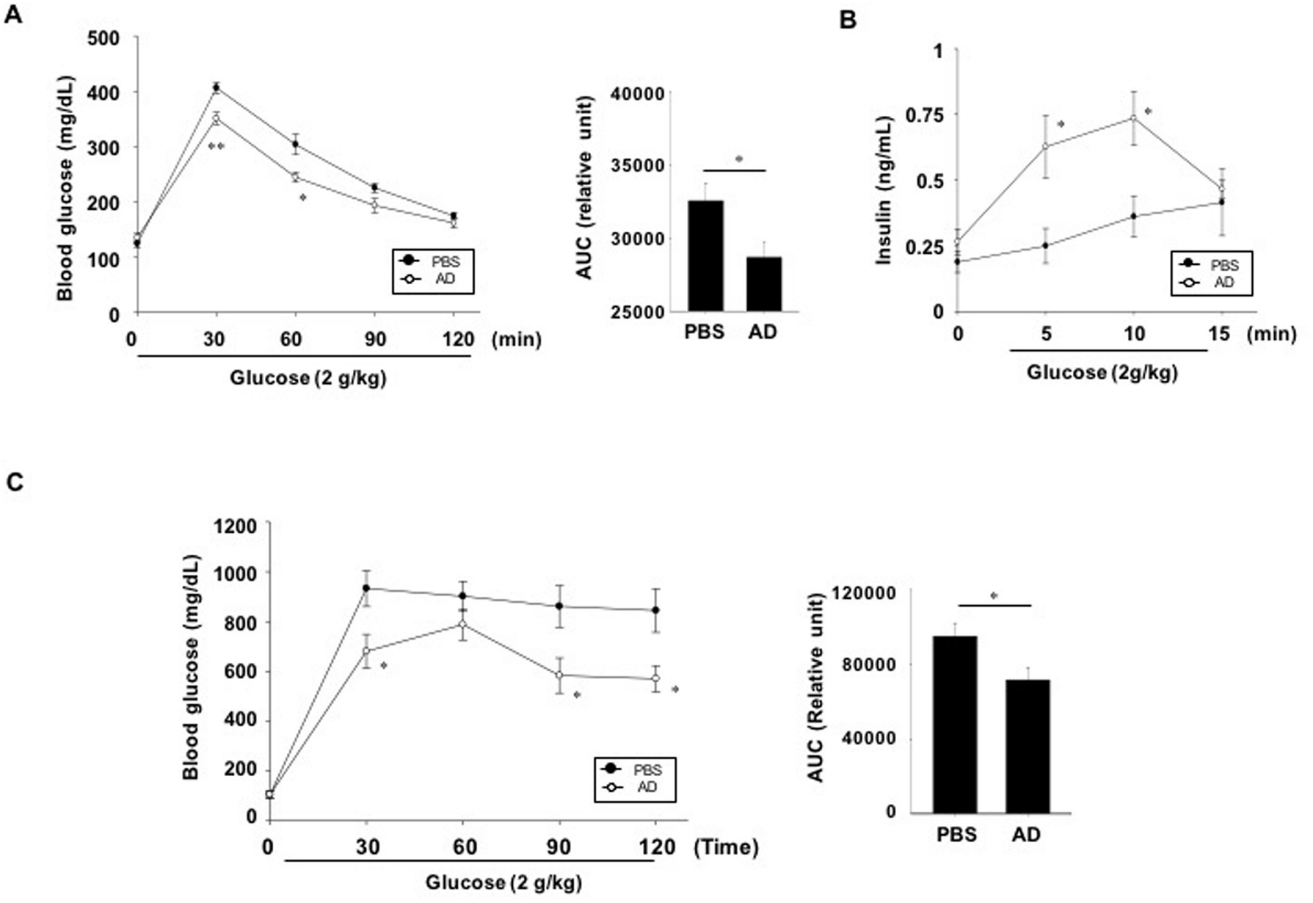
Effect of AD extracts on oral glucose tolerance and insulin secretion. (A, B) C57BL6 mice or C: db/db mice mice were not fed for 14 h and then AD extract (300 mg/kg body weight in PBS) or PBS (CON) was administered orally. (A, C) Thirty minutes later, oral glucose tolerance tests were performed. (B) Serum insulin levels were measured at 5, 10, and 15 min after glucose challenge. The area under the curve (AUC) for each glucose tolerance test was quantified (right panel of A and C). n = 7-9/group. Values are means ± SE. *p <0.05, **p <0.01 compared with control group.

To evaluate whether AD extracts improve glucose handling, oral glucose tolerance tests were performed. Blood glucose levels in AD extract-treated normal C57BL6 mice were significantly lower at 30 and 60 min following glucose intubation compared with the vehicle-treated mice group (Fig 2A, left). The area under the curve for the AD extract-treated normal C57BL6 mice was decreased 11.5% compared with the PBS -treated mice group (Fig 2A, right). To determine whether the improved glucose tolerance in AD extract-treated mice was a result of increased insulin secretion, we measured the serum insulin levels at 5, 10 and 15 min after oral glucose administration. The serum insulin levels in mice treated with AD extracts were significantly higher than those in vehicle-treated normal mice (Fig 2B). These results indicate that increased insulin secretion contributes to lowering the blood glucose in mice treated with AD extracts. To examine the glucose-lowering effects in diabetic mice, diabetic db/db mice were pretreated with AD extracts and oral glucose tolerance tests were performed. Glucose levels at each time point were significantly lower in AD extract treated mice than vehicle-treated control mice (Fig 2C, left) and area under the curve for the AD extract-treated mice was decreased 24.6% compared with vehicle-treated mice (Fig 2C, right).

### Effect of *n*-hexane fraction of AD extracts on secretion of GLP-1 and insulin *in vitro*

**Fig 3:**
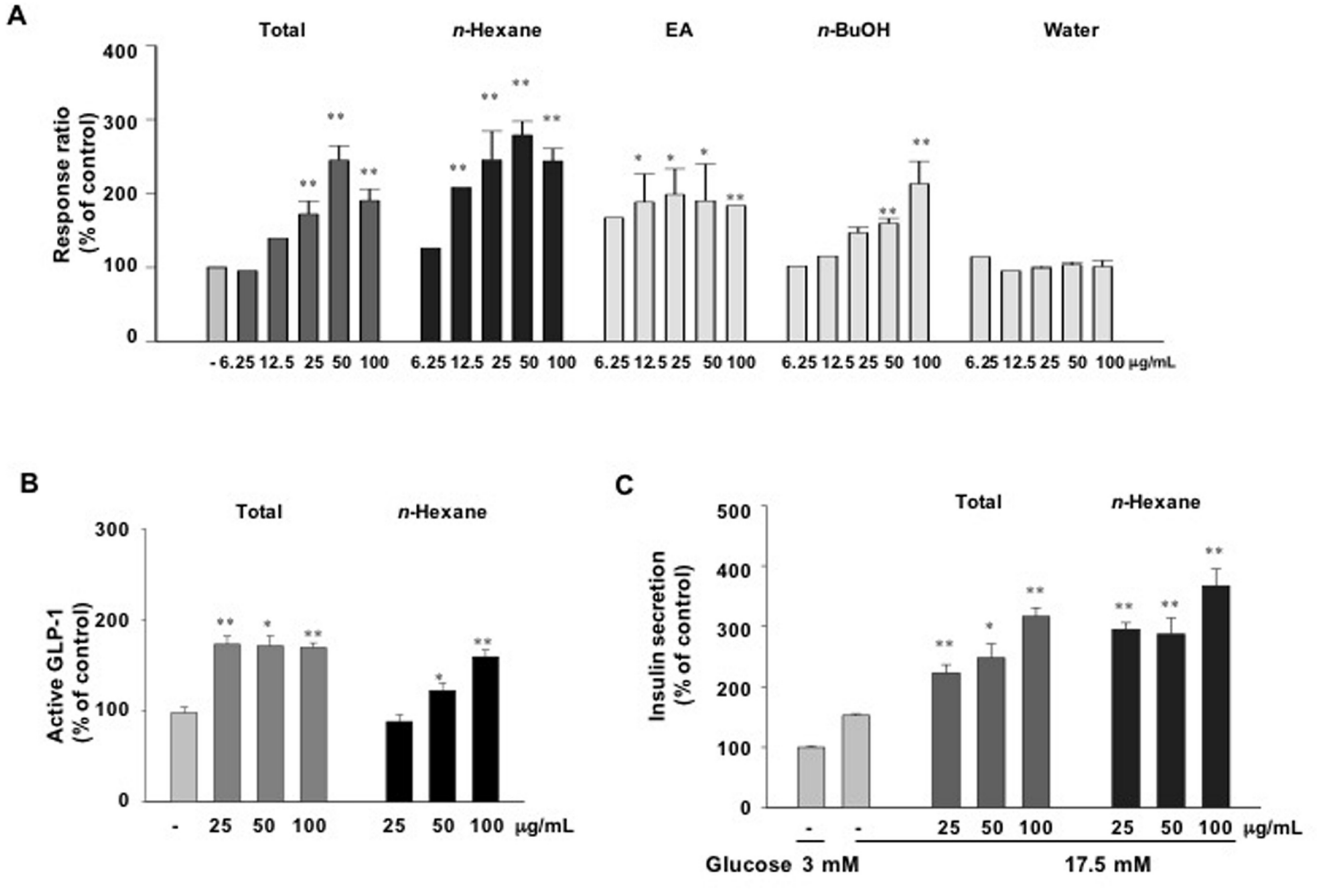
Agonistic and biological activities of various solvent fractions of AD extracts *in vitro*. (A) β-lactamase activity was measured in the GPR119-CRE-*bla* CHO-K1 cell line treated with the indicated concentrations of total crude (MeOH), *n*-hexane, ethyl acetate (EA), *n*-butanol (BuOH), or water extract for 5 h. (B) Active GLP-1 secretion in GLUTag cells treated with total or *n*-hexane extract. (C) Glucose-stimulated insulin secretion in INS-1 cells treated with total or *n*-hexane extract. n = 3-4/group. Values are means ± SE. *p <0.05, **p <0.01 compared with control group.

To evaluate the effect of various fractions of AD extracts on GPR119 activation, GPR119-CRE-*bla* CHO-K1 reporter cells were treated with different concentrations of *n*-hexane, ethyl acetate, *n*-butanol or water fractions. The *n*-hexane fraction of AD extracts induced the greatest increase in reporter activity (Fig 3A). As well, active GLP-1 levels in GLUTag cells (Fig 3B) and insulin secretion levels in INS-1 cells (Fig 3C) were significantly increased by the *n*-hexane fraction of AD extracts. These results suggest that the active components for GPR119 activation are contained in the *n*-hexane fraction of AD extracts.

### *In vivo* effect of the *n*-hexane fraction of AD extracts

**Fig 4:**
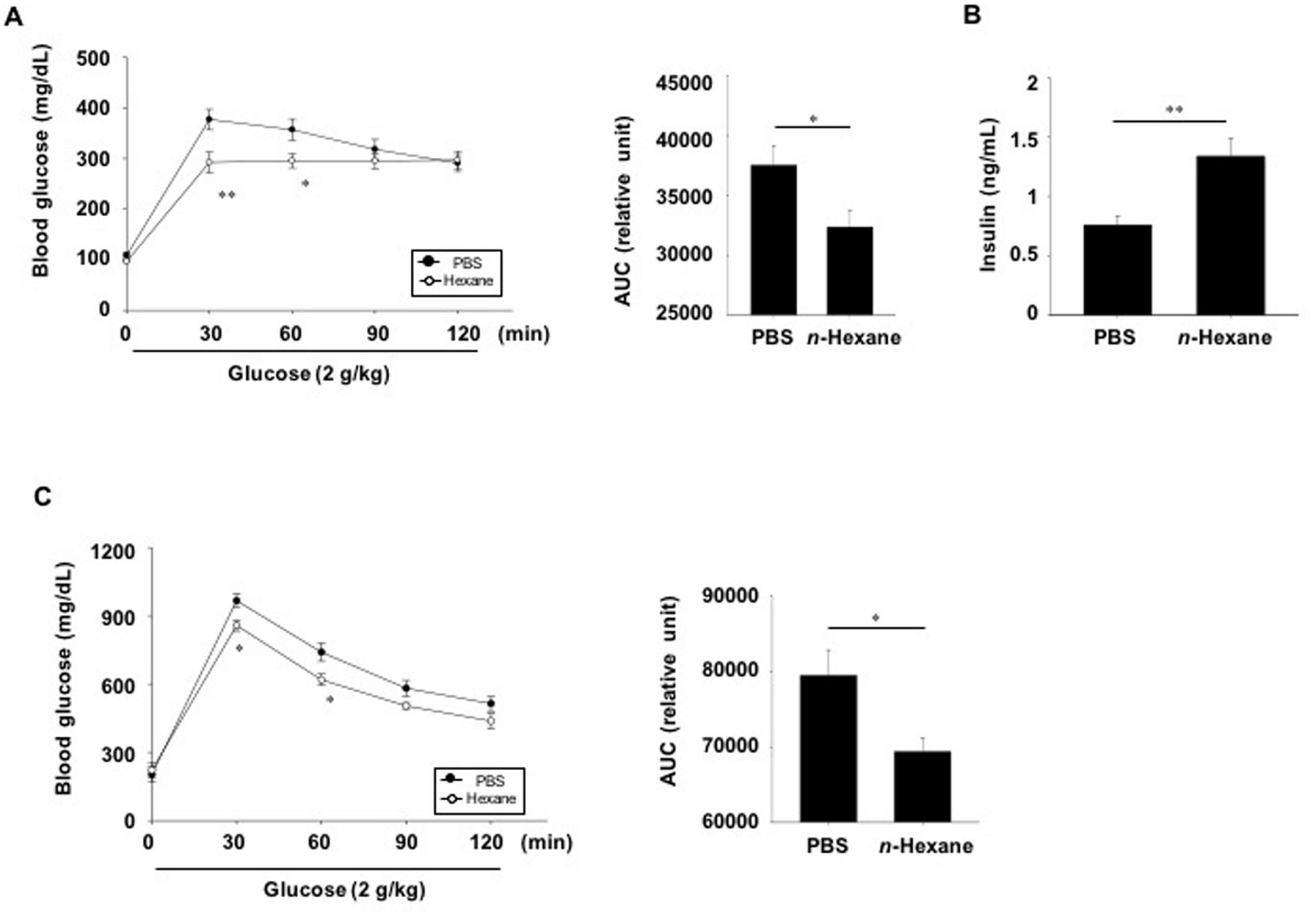
*In vivo* effect of *n*-hexane extract on glucose tolerance and insulin secretion. (A) C57BL6 mice (n = 15/group) and (C) db/db mice (n = 10-11/group) were not fed for 14 h and then *n*-hexane extract (300 mg/kg body weight in PBS) or PBS (CON) was administered orally. (A) Thirty minutes later, oral glucose tolerance tests were performed. The area under the curve (AUC) for glucose tolerance tests were quantified. (B) Plasma insulin levels were measured at 15 min after glucose challenge. Values are means ± SE. *p <0.05, **p <0.01 compared with PBS-treated control group.

To investigate the potential effects of the *n*-hexane fraction of AD extracts on glucose control, normal C57BL6 mice were given a single dose of the *n*-hexane extract (300 mg/kg), and glucose tolerance tests were performed 30 min later. Glucose levels were significantly lower at 30 and 60 min after oral glucose challenge in *n*-hexane fraction-treated mice compared with PBS -treated mice (Fig 4A). In addition, glucose-stimulated insulin secretion was significantly increased after a single dose of the *n*-hexane fraction of AD extracts (Fig 4B). We also performed oral glucose tolerance tests using diabetic db/db mice. Blood glucose levels in the *n*-hexane fraction-treated db/db mice were significantly lower 30 and 60 min following glucose injection than in the PBS treated db/db group (Fig 4C, left). The area under the curve of the *n*-hexane fraction-treated group decreased by 12.7% compared to that of the PBS-treated group (Fig 4C, right).

### Purification of active compounds from *n*-hexane fraction

**Fig 5:**
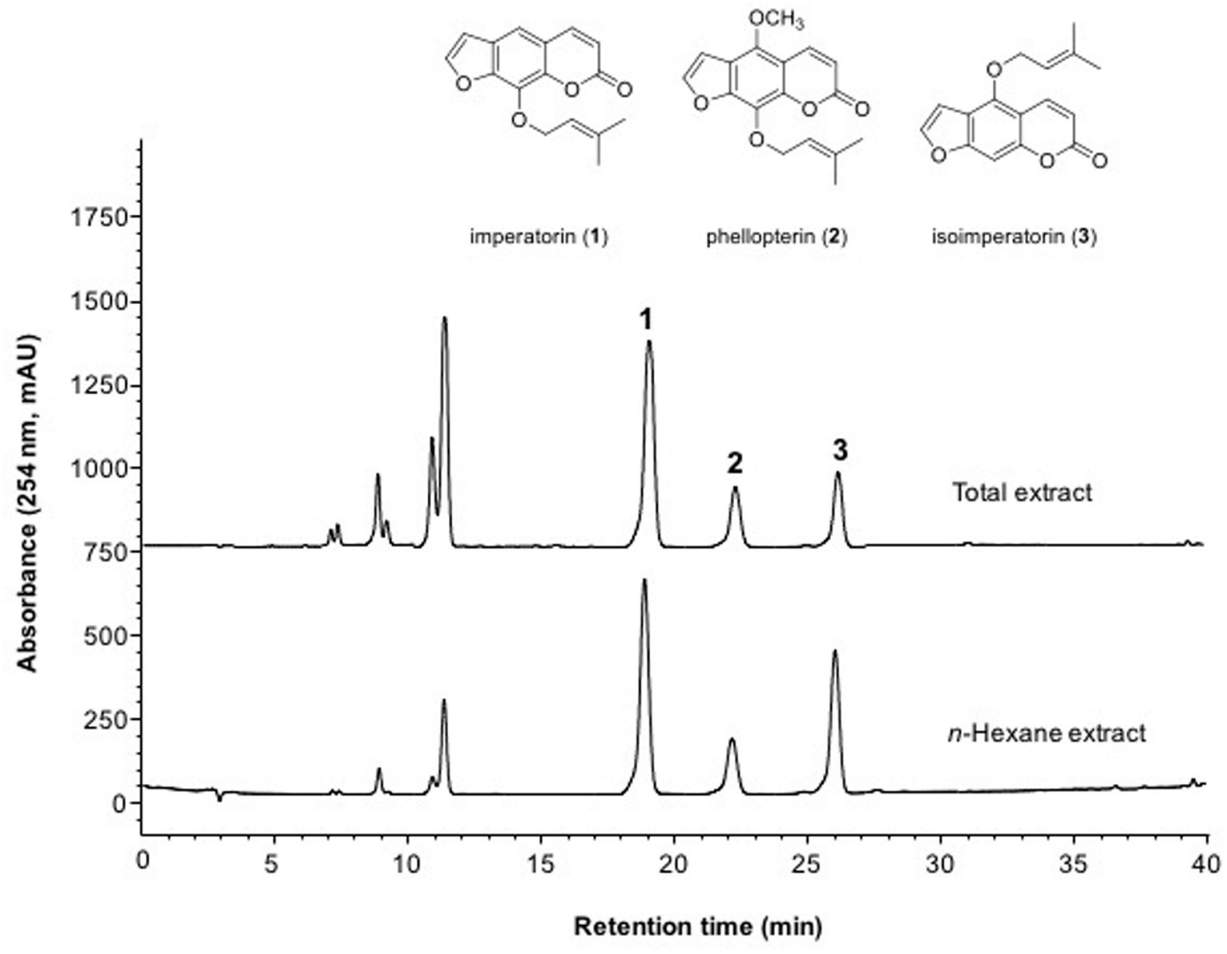
Chemical structures of isolated compounds from AD extract. HPLC chromatograms of AD total extract and *n*-hexane extract fractions. In the *n*-hexane fraction, imperatorin (**1**), phellopterin (**2**) and isoimperatorin (**3**) were identified by HPLC purification and ESI-MS, ^1^H and ^13^C NMR.

As *n*-hexane soluble fraction showed pharmacological activity, this fraction was further purified by preparative HPLC. Compounds **1**–**3** were collected between (**1**) 30.45–32.46 min, (**2**) 32.20–33.15 min and (**3**) 35.05–36.05 min, respectively (Fig 5). The chemical structures of isolated compounds were determined as imperatorin (**1**), phellopterin (**2**) and isoimperatorin (**3**) by comparison with previously published data [18].

### Effect of furanocoumarins isolated from *n*-hexane fraction as a GPR119 agonist

**Fig 6:**
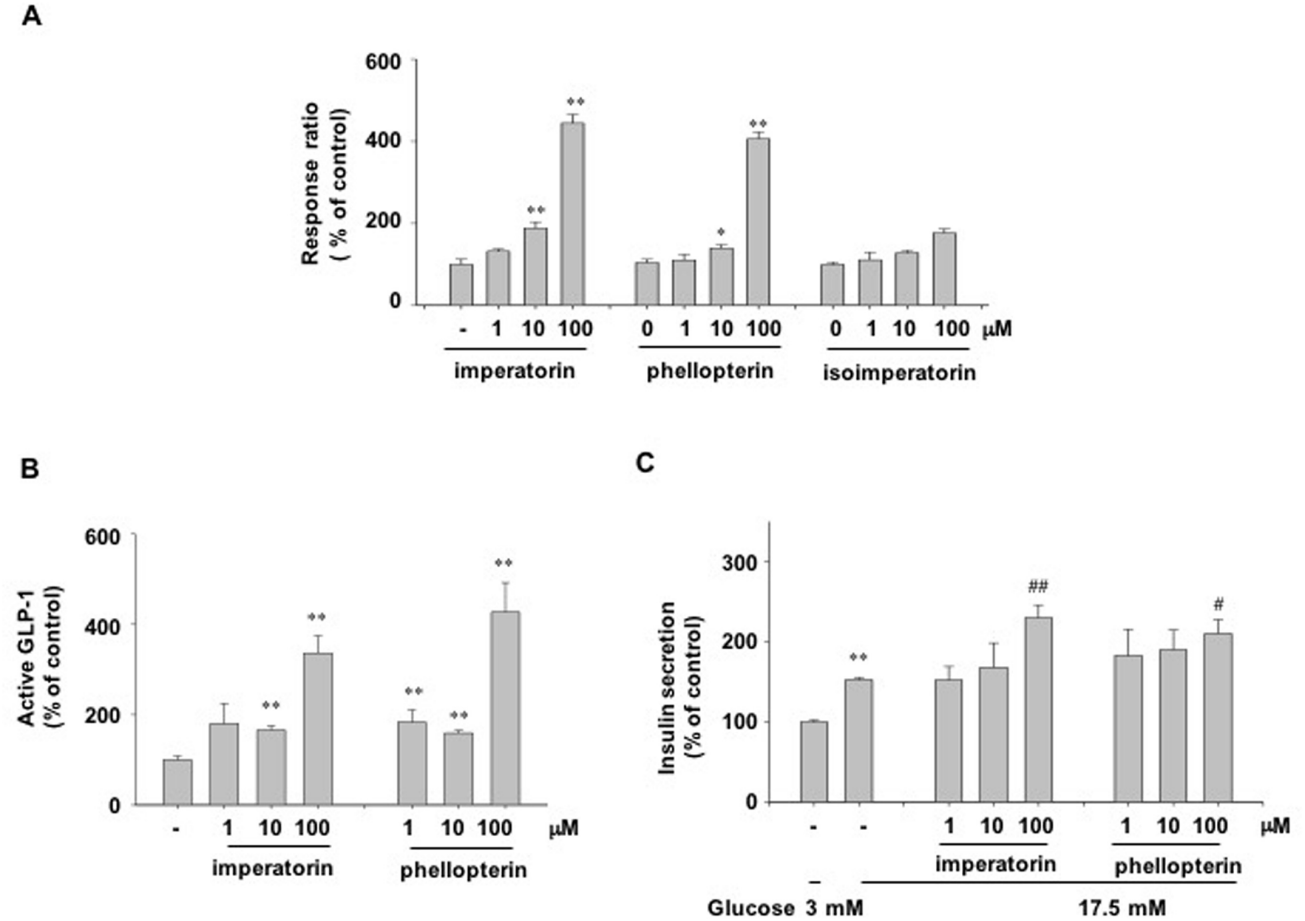
Agonistic and biological activities of furanocoumarins. (A) β-lactamase activity was measured in GPR119-CRE-*bla* CHO-K1 cells treated with the indicated concentrations of imperatorin, phellopterin or isoimperatorin for 5 h. (B) Active GLP-1 secretion in GLUTag cells. (C) Glucose-stimulated insulin secretion in INS-1 cells treated with the indicated concentrations of imperatorin or phellopterin in the presence of low (L, 3 mM) or high (H, 17.5 mM) glucose. n = 3-4/group. Values are means ± SE. *p <0.05, **p <0.01 compared with control group. #p<0.05, ##p<0.01 compared with vehicle-treated cells in the presence of high glucose.

We evaluated whether the compounds (imperatorin, phellopterin and isoimperatorin) obtained from the *n*-hexane fraction of AD extracts were GPR119 agonists. Treatment with imperatorin or phellopterin dose-dependently increased the reporter activity in GPR119-CRE-bla CHO-K1 cells, but treatment with isoimperatorin did not (Fig 6A). As well, imperatorin or phellopterin treatment significantly increased GLP-1 secretion in GLUTag cells (Fig 6B) and increased glucose-stimulated insulin secretion in INS-1 cells (Fig 6C).

### Effect of furanocoumarins on glucose tolerance in C57BL6 mice and db/db mice

**Fig 7:**
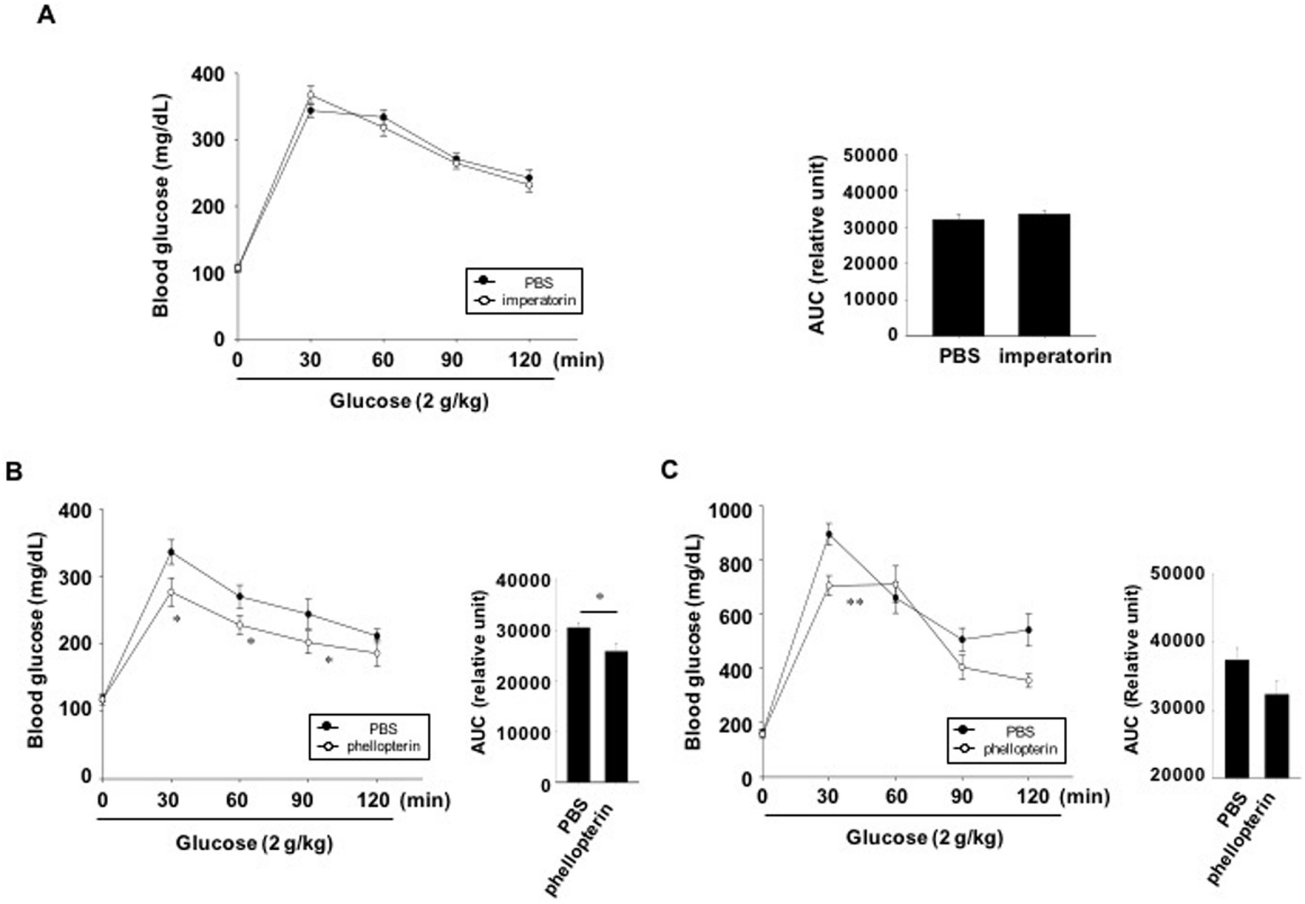
*In vivo* effect of imperatorin and phellopterin on glucose tolerance. (A, B) C57BL6 mice were not fed for 14 h and then imperatorin (30 mg/kg, in PBS, n = 9) or phellopterin (30 mg/kg in PBS, n = 7–8) was administered orally. (C) Diabetic db/db mice were not fed for 14 h and then phellopterin (30 mg/kg in PBS, n = 6) was administered orally. PBS was administered as a control (CON). Thirty minutes later, oral glucose tolerance tests were performed. The area under the curve (AUC) for each glucose tolerance test was quantified. n = 8-12/group. Values are means ± SE. *p <0.05, **p <0.01 compared with PBS treated group.

To examine whether imperatorin or phellopterin improves glucose tolerance, we performed oral glucose tolerance tests after pretreatment with imperatorin or phellopterin in C57BL6 mice. Blood glucose levels in the phellopterin-, but not imperatorin-treated mice were significantly lower at 30, 60 and 90 min following glucose administration compared with the vehicle-treated mice group (Fig 7A and 7B). These results suggest that phellopterin, obtained from AD extract may be one of the candidates for GPR119 agonistic actions. We then assessed whether phellopterin could improve glucose tolerance in diabetic db/db mice. Blood glucose levels in phellopterin-treated mice were significantly lower than those in PBS-treated mice 30 min after glucose injection (Fig 7C).

### Glucose lowering effect of multiple doses of AD extracts or *n*-hexane fractions in db/db mice

**Fig 8:**
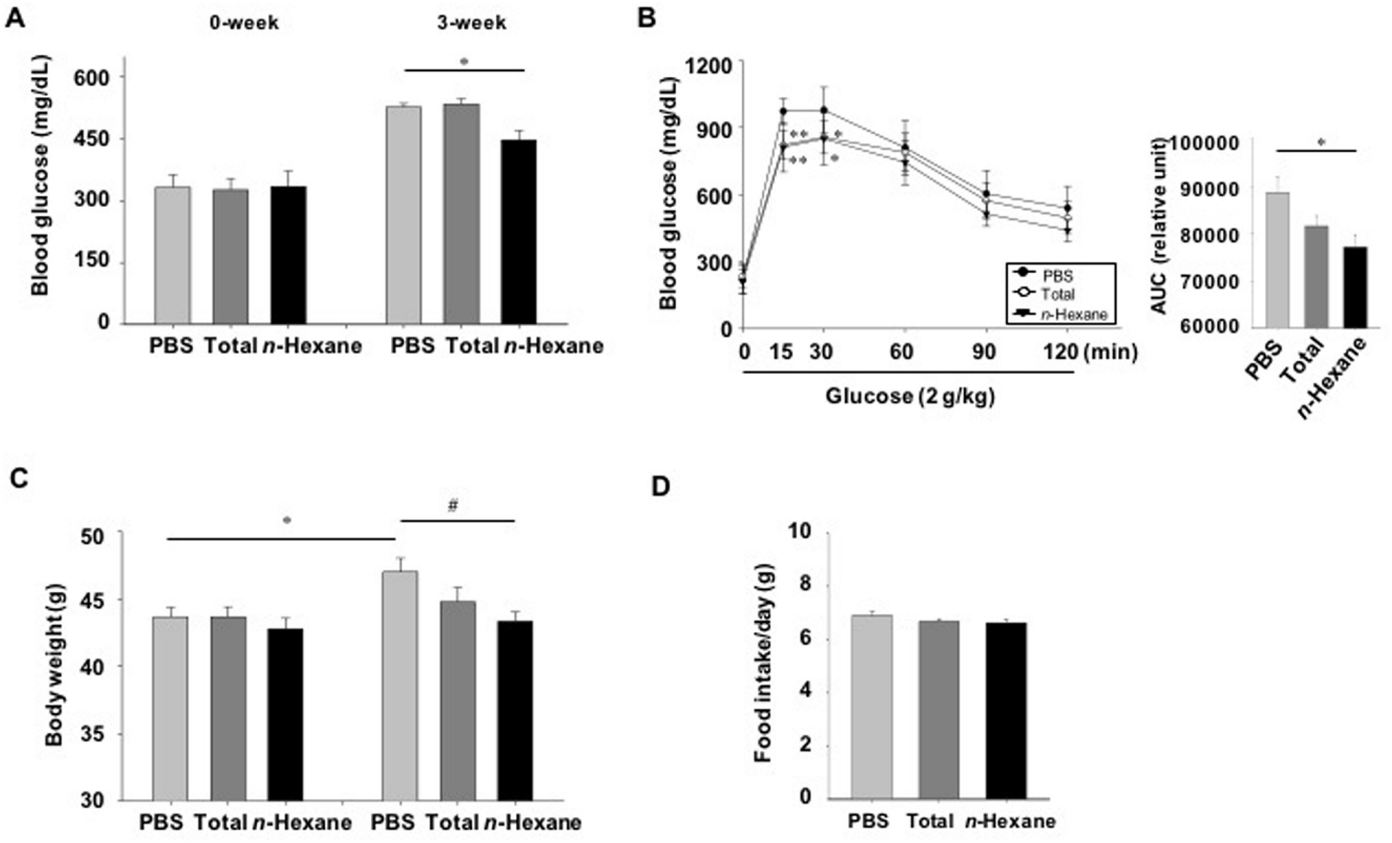
Long-term effect of AD extracts or *n*-hexane fractions in lowering glucose in diabetic db/db mice. Diabetic db/db mice were orally administered AD extracts, *n*-hexane fractions (300 mg/kg body weight in PBS), or PBS daily for three weeks. (A) Blood glucose levels were measured three weeks after treatment, and (B) oral glucose tolerance tests were performed (n = 9–10). The area under the curve (AUC) for each glucose tolerance test was quantified. (C) Body weights were monitored after three weeks of treatment. (D) Food intake was measured weekly. Values are the average weight of food consumed/mouse/day. Values are means ± SE. *p < 0.05, **p < 0.01 compared with the PBS-treated group. #p < 0.05 compared with the PBS-treated group at three weeks of treatment.

To investigate whether long-term treatment with AD extracts or *n*-hexane fractions could reduce blood glucose levels, we measured blood glucose levels in diabetic db/db mice three weeks after treatment. Blood glucose levels significantly decreased in *n*-hexane fraction-treated mice compared to those in PBS-treated mice (Fig 8A). However, AD extract-treatment had no glucose lowering effect (Fig 8A). We then performed oral glucose tolerance tests three weeks after treatment. Blood glucose levels in *n*-hexane fraction-treated mice were significantly lower than those in PBS-treated mice at 15 and 30 min after glucose loading (Fig 8B, left panel). The area under the curve for the AD extract- and *n*-hexane fraction-treated mice decreased compared to that for PBS-treated mice, by 8% and 13%, respectively (Fig 8B, right panel). Body weight gain significantly decreased in AD extract-treated (before: 43.7g ± 0.7, after: 44.8 g ± 1.0) and n-hexane fraction-treated mice (before: 42.8g ± 0.7, after: 43.3 g ± 0.6) compared to that of PBS-treated mice (before: 43.7g ± 0.7, after: 47.3g ± 1.0); however, food consumption was not significantly different among the PBS, AD extract, or *n*-hexane groups (Fig 8C, right).

## Discussion

Pancreatic β-cell function and mass declines during the progression of type 2 diabetes, and there is a growing need for remedies to support β-cell function [19]. Emerging evidence suggests that several therapeutic agents can protect the function of the pancreatic β-cells. GLP-1 is an incretin hormone secreted from entero-endocrine cells in response to ingesting nutrients. When GLP-1 binds to its cognate receptor, glucose-stimulated insulin secretion is enhanced in pancreatic β-cells. Moreover GLP-1 improves the survival of β-cells and prevents their apoptosis [9]. Because GLP-1 mimetics are administered by injection, other agents that can be administered orally must be developed.

GPR119 agonists have been highlighted as ideal therapeutic agents for type 2 diabetes because they increase GLP-1 secretion and can be orally administered [10, 20, 21]. GPR119 is coupled to the adenyl cyclase/cyclic AMP signaling pathway via Gαs protein [22]. In our study, the β-lactamase reporter gene, containing the cAMP responsive element, was used for high-throughput screening of 1500 natural products. Of the 30 hits found, AD extract was chosen based on its ability to increase intracellular cAMP levels in GLUTag and INS-1 cells. It has been reported that other GPR119 agonists enhance active GLP-1 secretion in L-cells and glucose-stimulated insulin secretion in pancreatic β-cells [10, 11]. In our study, AD extract treatment had a similar effect: AD extract significantly increased active GLP-1 secretion and insulin release in GLUTag cells and INS-1 cells, respectively. When we performed oral glucose tolerance tests after oral administration of a single dose AD extract in C57BL6 mice, glucose levels were significantly decreased and insulin was increased at a dose of 300 mg/kg compared with vehicle (PBS)-treated control mice. Oral administration of AD extract in diabetic db/db mice also improved glucose tolerance compared with vehicle-treated control mice. These results suggest that AD extract may have GPR119 agonistic actions and control blood glucose levels by enhancing glucose-stimulated insulin secretion.

To find the active fraction from AD extract, the total methanol extract was partitioned into *n*-hexane, ethylacetate, *n*-butanol and water fractions. Although the *n*-hexane, ethylacetate, and *n*-butanol extracts possessed GPR119-dependant cAMP accumulation ability, the *n*-hexane fraction seemed to have the most potent activity in the GPR119-CRE-*bla* CHO-K1 cell line. The crude methanol extract showed more potent effect on GLP-1 secretion than the *n*-hexane fraction, whereas the *n*-hexane fraction showed more potent effect on insulin secretion than crude methanol extract. In addition, long-term treatment (3 weeks) of db/db mice with an n-hexane fraction showed a stronger anti-diabetic effect, as evidenced by a decrease of blood glucose levels and improved glucose tolerance, than that of compared with the AD extract-treated mice. This discrepancy may be due to the various active components contained in the different solvent extract.

To further identify the active component of the *n*-hexane extract, this fraction was further purified by preparative HPLC followed by ESI-MS, ^1^H and ^13^C NMR. The *n*-hexane extract contained byakangelicol, oxypeucedanin, imperatorin, phellopterin and isoimperatorin. Among these furanocoumarins, imperatorin is known to inhibit carbohydrate metabolizing enzymes, possibly having an anti-diabetic effect [23]. However, the anti-diabetic functions of other furanocoumarins have not been defined. Because of the large amounts of imperatorin, phellopterin and isoimperatorin in the *n*-hexane fraction of AD extract, we examined these compounds first. Imperatorin and phellopterin had GPR119 agonistic activity in the reporter cell line, but isoimperatorin did not. GLP-1 secretion and glucose-stimulated insulin secretion levels were increased in both imperatorin- or phellopterin-treated cells *in vitro*. However, an *in vivo* effect on the improvement of glucose tolerance in normal C57BL6 mice and db/db mice was only observed after phellopterin treatment, but not after imperatorin treatment. Phellopterin was also effective for the improvement of glucose tolerance in db/db mice. Different *in vivo* efficacies might be due to differences in the absorption, and metabolism of the active components. In addition, the effects of byakangelicol and oxypeucedanin as a putative GPR119 agonists need to be examined.

## Conclusion

In conclusion, we identified an AD extract and its active component, phellopterin, as a novel anti-diabetic agent that is capable of increasing insulin secretion and improving glucose tolerance *in vivo* through GPR119 activation.

## Supporting Information

S1 Fig HPLC chromatograms of *n*-hexane extract of *Angelica dahurica*, and purified compounds (1–3). HPLC conditions were described in the Materials and methods section. Purity of isolated compounds (**1**–**3**) was over 95% based on the peak area.
(TIF)

S2 Fig ^1^H NMR spectrum of imperatorin (1) (400 MHz, CDCl_3_). (TIF)

S3 Fig ^1^H NMR spectrum of phellopterin (2) (400 MHz, CDCl_3_). (TIF)

S4 Fig ^1^H NMR spectrum of isoimperatorin (3) (400 MHz, CDCl_3_). (TIF)
