# Supplementary figures and images for: Angelica dahurica Extracts Improve Glucose Tolerance through the Activation of GPR119
Source: PLoS One. 2016 Jul 8;11(7):e0158796. doi: 10.1371/journal.pone.0158796 (PMC4938581; doi:10.1371/journal.pone.0158796)

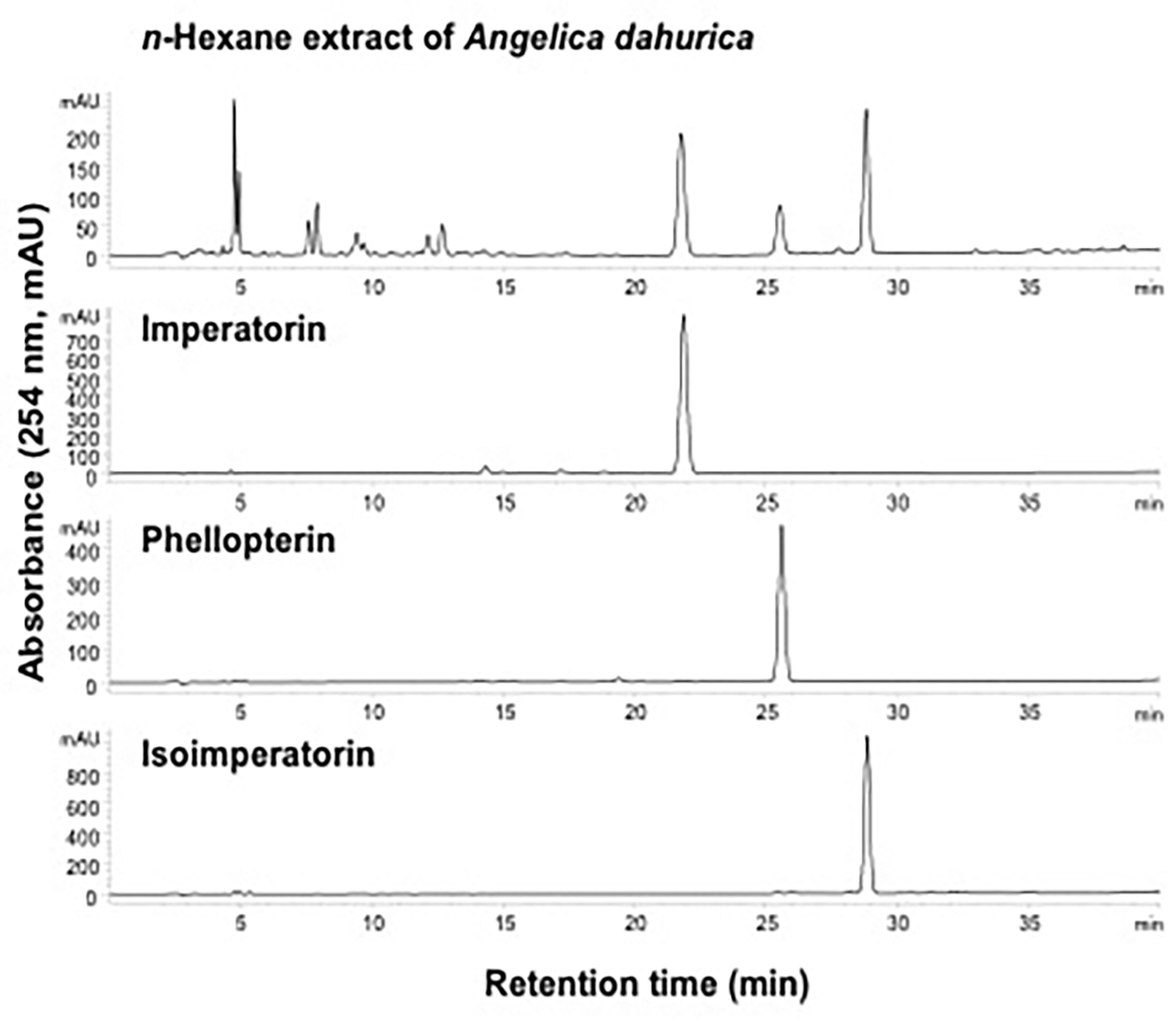

Supplement: S1 Fig — HPLC conditions were described in the Materials and methods section. Purity of isolated compounds (1–3) was over 95% based on the peak area. (TIF) [file pone.0158796.s001.tif]

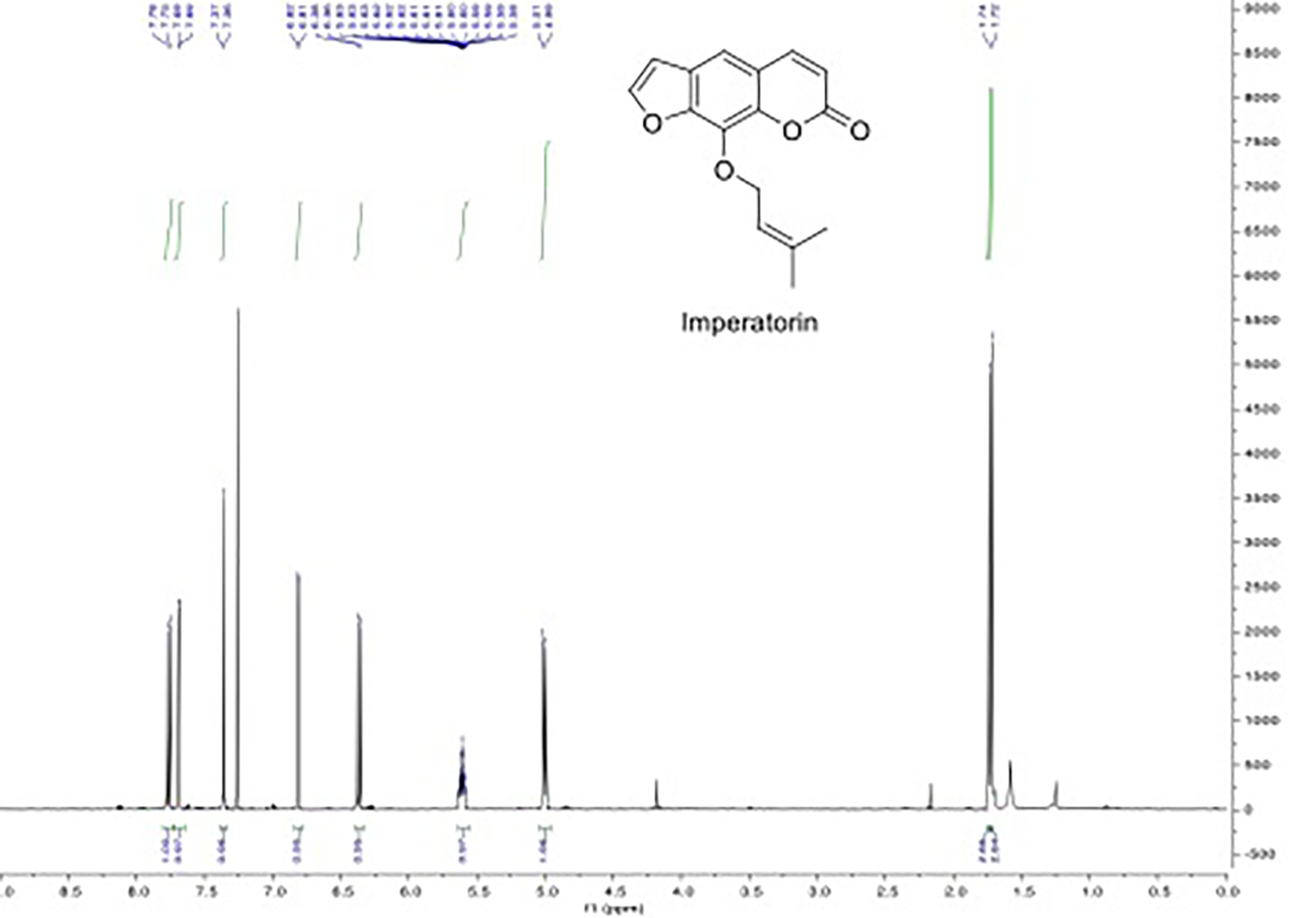

Supplement: S2 Fig — (TIF) [file pone.0158796.s002.tif]

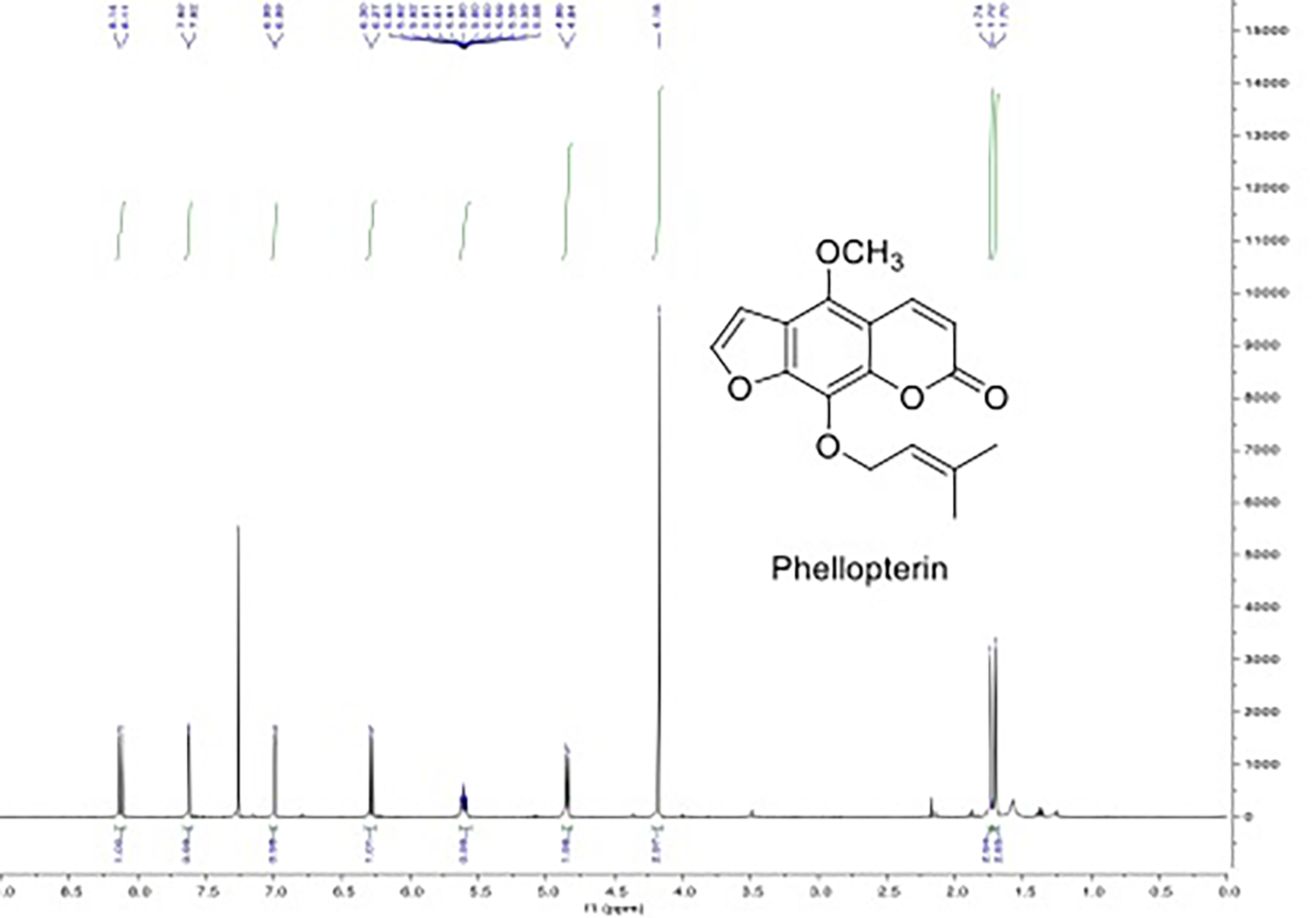

Supplement: S3 Fig — (TIF) [file pone.0158796.s003.tif]

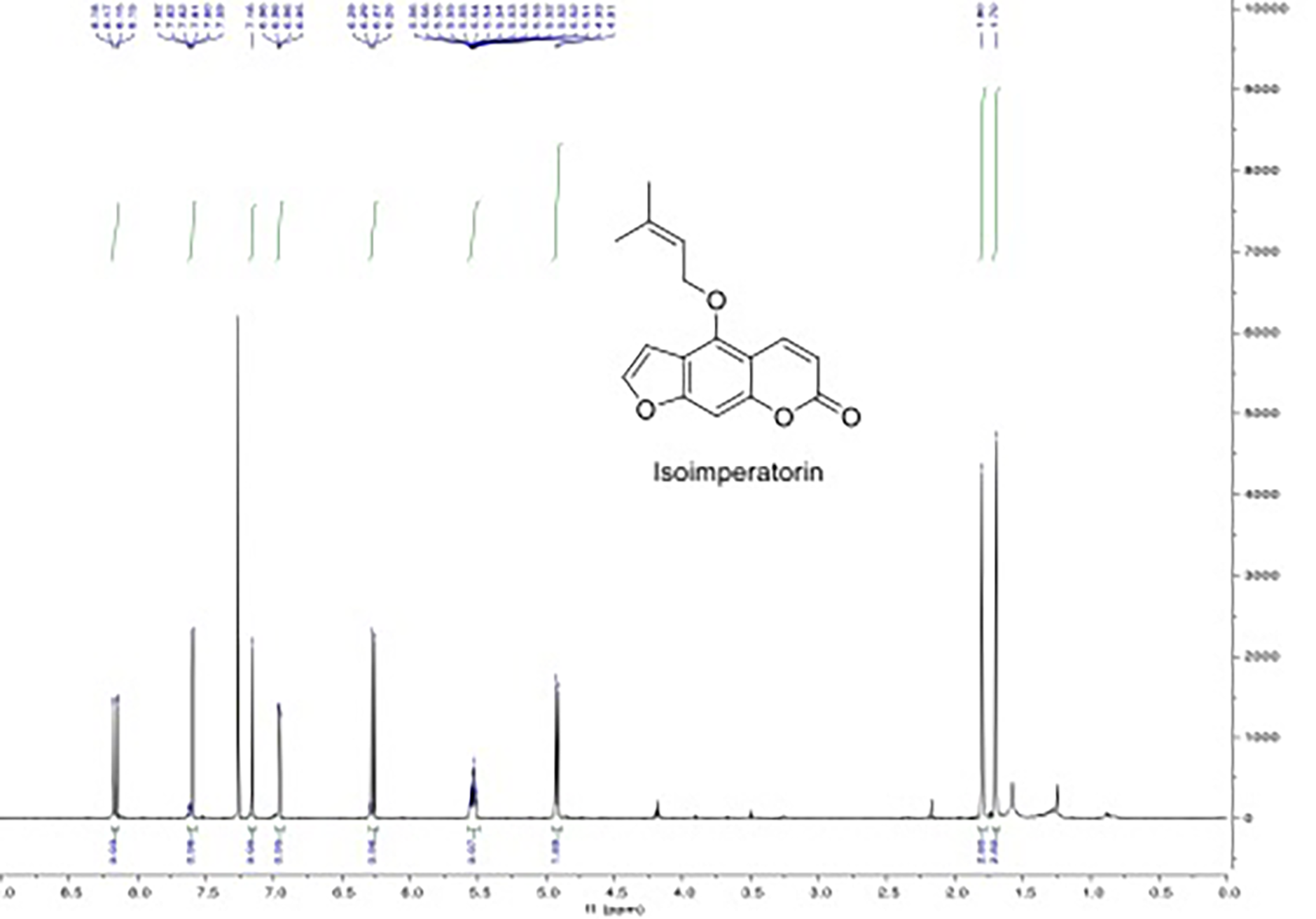

Supplement: S4 Fig — (TIF) [file pone.0158796.s004.tif]
